# A histochemical reporter system to study extracellular ATP response in plants

**DOI:** 10.3389/fpls.2023.1183335

**Published:** 2023-06-02

**Authors:** Joel M. Sowders, Kiwamu Tanaka

**Affiliations:** ^1^ Department of Plant Pathology, College of Agricultural, Human, and Natural Resource Sciences, Washington State University, Pullman, WA, United States; ^2^ Molecular Plant Sciences Program, Washington State University, Pullman, WA, United States

**Keywords:** transcriptional histochemical reporters, damage-associate molecular patterns (DAMPs), purinoceptor P2K1, purinergic sigaling, extracellular ATP (eATP), signal transduction, root tip

## Abstract

When cells experience acute mechanical distress, they release ATP from their cellular compartment into the surrounding microenvironment. This extracellular ATP (eATP) can then act as a danger signal—signaling cellular damage. In plants, cells adjacent to damage detect rising eATP concentrations through the cell-surface receptor kinase, P2K1. Following eATP perception, P2K1 initiates a signaling cascade mobilizing plant defense. Recent transcriptome analysis revealed a profile of eATP-induced genes sharing pathogen- and wound-response hallmarks—consistent with a working model for eATP as a defense-mobilizing danger signal. To build on the transcriptional footprint and broaden our understanding of dynamic eATP signaling responses in plants, we aimed to i) generate a visual toolkit for eATP-inducible marker genes using a β-glucuronidase (GUS) reporter system and ii) evaluate the spatiotemporal response of these genes to eATP in plant tissues. Here, we demonstrate that the promoter activities of five genes, *ATPR1*, *ATPR2*, *TAT3*, *WRKY46*, and *CNGC19*, were highly sensitive to eATP in the primary root meristem and elongation zones with maximal responses at 2 h after treatment. These results suggest the primary root tip as a hub to study eATP-signaling activity and provide a proof-of-concept toward using these reporters to further dissect eATP and damage signaling in plants.

## Introduction

Although typically regarded as an intracellular metabolite and potential energy storage molecule, the high concentration of ATP within the cell (mM) and its relatively low extracellular concentration (~nM) make it a potent signal molecule. In both animals and plants, wounding (Song et al., 2006), physical stimulation (Sauer et al., 2000; Jeter et al., 2004), and microbe interactions (Torres et al., 2008; Nizam et al., 2019) cause ATP to be released from its cellular container into the extracellular space. Once in the extracellular space, ATP becomes a danger signal (Tanaka et al., 2014). As a signal, extracellular ATP (eATP) belongs to a group of defense molecules known as damage-associated molecular patterns or DAMPs (Tanaka and Heil, 2021).

The first plant receptor for extracellular ATP identified is a plasma membrane-localized legume-type lectin receptor kinase (LecRK), also known as P2 Kinase 1, or P2K1, which was identified from the *does not respond to nucleotides1* (*dorn1*) mutant (Choi et al., 2014). P2K1 elicits secondary messenger reactive oxygen species (ROS) and cytosolic calcium (Ca^2+^) influx following eATP perception (Jeter et al., 2004; Song et al., 2006; Demidchik et al., 2009; Tanaka et al., 2010; Choi et al., 2014; Chen D. et al., 2017; Wang et al., 2018). P2K1-dependent eATP signaling impacts a diverse range of plant–pathogen interactions, including those involving viruses, bacteria, fungi, oomycetes, nematodes, and even insects (Bouwmeester et al., 2011; Bouwmeester et al., 2014; Wang et al., 2014; Balagué et al., 2017; Kumar et al., 2020; Jewell et al., 2022). Recently, it was reported that the indolic glucosinolate pathway can be induced by eATP in *Arabidopsis thaliana* (*Arabidopsis*), serving as a biochemical defense against pathogens and pests (Jewell et al., 2022). Although the impact of eATP signaling in plants is well documented, characterizing the molecular mechanisms that connect early eATP signaling activities by the receptor P2K1 (i.e., ROS and Ca^2+^) to plant immunity is a work in progress.

Recent headway has been made by revealing P2K1-dependent eATP-responsive transcriptional activities, which showed expression of many genes involved in wound and defense response that are linked to jasmonic acid (JA) signaling, a key plant defense hormone—suggesting that eATP could employ JA-mediated signal amplification (Tripathi et al., 2018; Jewell et al., 2019). To gain a deeper understanding of P2K1-mediated signaling activity through the eATP-induced transcriptome, we created a kit of ATP-responsive marker tools using the β-glucuronidase (GUS) reporter system in *Arabidopsis*. We hypothesized that evaluating the expression patterns of eATP-responsive markers would reveal previously unknown spatial and temporal aspects of eATP signaling in plants. Additionally, we expected that eATP-responsive reporter tools would allow for the visualization of eATP-responsive signaling with a high resolution of spatiotemporal precision, for example, making them useful for high-throughput forward genetic screens. Five highly responsive eATP marker genes were selected from the published transcriptome (Jewell et al., 2019) to generate promoter::reporters for *TAT3*, *WRKY46*, *CNGC19*, and two uncharacterized genes, which have been named *ATP RESPONSIVE 1* (*ATPR1*; AT5G19110) and *ATP RESPONSIVE 2* (*ATPR2*; AT3G44870), respectively. Each of the five markers displayed maximal eATP-responsive expression in the root tip of the *Arabidopsis* seedlings. These tools will allow us to unlock additional insights into the mechanisms underlying eATP-mediated signaling.

## Methods

### Plant growth

*Arabidopsis* plants of wild type (ecotype Columbia-0 or Col-0), *dorn1-3* (Choi et al., 2014), and transgenic lines were each surface- sterilized and sown on a solid medium containing half-strength Murashige and Skoog (MS) (Murashige and Skoog, 1962) with vitamins (MSP09; Casson, Smithfield, UT, USA), 1% (w/v) sucrose, and 1% (w/v) agar Daishin (RPI, Mt. Prospect, IL, USA) and buffered in 0.05% (w/v) MES-KOH (pH 5.7) in a square petri dish. Seeds were cold- stratified in the dark for 3 days at 4°C and grown vertically in a 22°C growth chamber (CMP6010; Conviron, Winnipeg, MB, Canada) with a 12-h photoperiod (~100 µmol photons m^−2^ s^−1^).

### ATP treatment

Working ATP stock solution (100 mM) was prepared by dissolving ATP (Sigma-Aldrich, St. Louis, MO, USA) in 2 mM of MES. The pH was adjusted to 5.7 with KOH, and the solution was filter-sterilized and stored in aliquots at −20°C indefinitely. For the GUS reporter and RT-qPCR experiments, *Arabidopsis* plants were grown for 5 or 7 days, respectively. After the vertical growth period, the seedlings were transferred to six-well plates (15–20 seedlings per well) containing half-strength MS (as above) without agar (“liquid media”). Once transferred to a liquid medium, the seedlings were returned to the growth chamber to equilibrate overnight. The following day, the medium in each well was replaced with 1 ml of liquid medium (mock) or ATP solution (in liquid medium) at a final concentration of 500 μM. For the RT-qPCR experiments, the seedlings were blotted dry and snap-frozen in liquid nitrogen 30 min after treatment. In the GUS reporter experiments, treatment durations were variable and the seedlings were harvested and fixed in ice-cold 90% (v/v) acetone.

### Transgenic material

The transgenic *Arabidopsis* lines *pP2K1::GUS* (Cho et al., 2017) and *proCNGC19::GUS* #4 (Kugler et al., 2009) were graciously provided by Dr. Gary Stacey at the University of Missouri and Dr. Petra Dietrich at the University of Erlangen, Germany, respectively. To construct GUS reporters for the other eATP marker genes [*CML39* (AT1G76640), *TAT3* (AT2G24850), *WRKY46* (AT2G46400), *ATPR1* (AT5G19110), and *ATPR2* (AT3G44870)], 800 –2,500-bp non-coding regions upstream of each gene were amplified from genomic DNA using primers designed with the 5′-tails containing restriction sites of *Hin*dIII and *Nco*I, respectively (**Table S1**). Once amplified, the promoter fragments were ligated into pCAMBIA1305.2, yielding a promoter linked to the β-glucuronidase (GUS) reporter. After the transformation and propagation of plasmids in *E scherichia coli* (DH5α), the promoter::GUS constructs were validated by Sanger sequencing (Elim Biopharmaceuticals, Inc., Hayward, CA, USA) and introduced to *Agrobacterium* (GV3101 pMP90) for plant transformation (Koncz and Schell, 1986). *Arabidopsis* plants (Col-0 or *dorn1-3*) were transformed using the floral dip method (Clough and Bent, 1998) and selected in the next generation on hygromycin-containing media to obtain independent T3 homozygous lines. All transgenic lines created are listed in **Table S2**.

### Histochemical staining and imaging

After ATP treatment, the transgenic *Arabidopsis* plants expressing GUS reporter constructs were harvested in ice-cold 90% (v/v) acetone for 20 min. The seedlings were blotted dry, washed once with sterile deionized water to remove residual acetone, and returned to a clean six-well plate. In the dark, the seedlings were incubated with 3 ml of the GUS staining solution (2 mM of potassium ferricyanide, 2 mM of potassium ferrocyanide, 0.25% (v/v) Triton X-100, 50 mM of sodium phosphate pH 7.2, 1 mM of X-Gluc), placed under vacuum at room temperature for 20 min, and transferred to a 37°C incubator for an additional 60 min. The GUS staining solution was replaced with 3 ml of 70% ethanol, and the samples were heated to 70°C for 30 min. Afterward, fresh 70% (v/v) ethanol was added and left overnight at room temperature. The next day, the samples were heated again to 70°C for 30 min, and the ethanol was replaced with fresh 70% ethanol. This was repeated a third time the following day with 90% (v/v) ethanol and finally replaced with 100% (v/v) ethanol. The samples were then stored in 100% (v/v) ethanol at 4°C until imaged, no more than 48 h later. Primary root zones were defined as previously described (Beemster and Baskin, 1998; Dello Ioio et al., 2008; Perilli et al., 2012; Petricka et al., 2012). Microscopic images were obtained with an inverted phase-contrast microscope (Leica DMI6000) or stereo microscope (Leica MZ10 F).

### RT-qPCR

After the samples were snap-frozen in liquid nitrogen, they were stored in a −80°C freezer. Frozen tissue was homogenized using the Mini-Beadbeater™ (BioSpec Products, Bartlesville, OK, USA), and RNA isolation was performed with the Quick-RNA isolation kit (Zymo Research, Irvine, CA, USA) according to the manufacturer’s protocols. RNA concentration was measured with an Eppendorf μCuvette® G1.0 on the BioPhotometer® D30 (Eppendorf, Hamburg, Germany), and 1 μg of RNA was used for cDNA synthesis (iScript; Bio-Rad, Hercules, CA, USA). The cDNA was 10-fold diluted with sterile deionized water, of which 2 μl was used in a 20-μl SYBR Green reaction mix (SsoAdvanced; Bio-Rad). All reactions were run in a CFX96 thermocycler (Bio-Rad). Gene expression was normalized to *PP2A* and *SAND* with similar results (Czechowski et al., 2005; Zhang et al., 2015). All primers used are listed in **Table S1**.

### Image processing

Adobe Photoshop 2020 (v21.2.10.118) was used for processing microscope image data. To improve visual interpretation, post-capture white balancing and removal of background noise were done to provide a fair comparison between experiments performed on different dates. All image data presented in this study are representative depictions of at least two and, in most cases, three independent stable transgenic lines (not including lines previously published) and have been observed across hundreds of samples in no less than five independent experiments.

## Results

### Spatial expression of the eATP receptor P2K1

**Figure 1:**
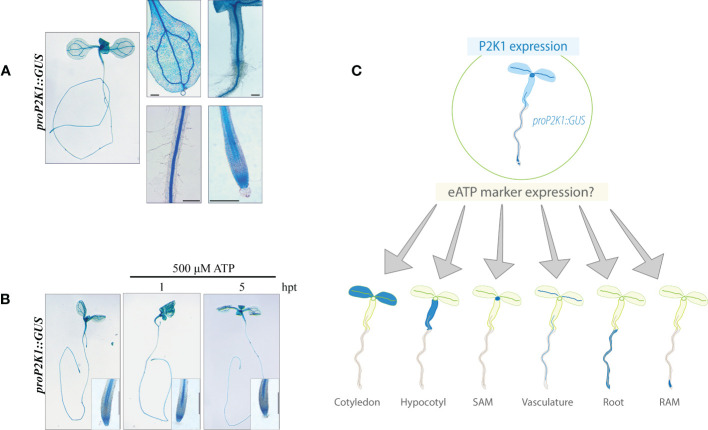
P2K1 promoter activity is not responsive to ATP treatment. **(A)** Histochemical β-glucuronidase (GUS) staining in *pP2K1::GUS* transgenic plants. The *P2K1* promoter is active in various tissues of 7-day-old seedlings. **(B)** Seven-day-old *pP2K1::GUS* seedlings were incubated with MS or MS plus 500 μM of ATP for 1 or 5 h post-treatment (hpt). Scale bar = 300 μm. **(C)** Graphical research question: What is the spatiotemporal expression pattern of eATP marker genes? RAM, root apical meristem; SAM, shoot apical meristem.

The expression patterns of the extracellular ATP receptor P2K1 have previously been described using the GUS reporter line *pP2K1::GUS* (Cho et al., 2017). We re-evaluated the expression of *P2K1* in our lab using the same reporter line. In 4-day-old plants, P2K1 was previously shown to be highly expressed throughout various tissue types (Cho et al., 2017), while, here, the expression in 7-day-old plants was confined to leaf mesophyll and vasculature, root junction, root vasculature, and root tip tissues (**Figure 1A**). Our findings also revealed that the expression pattern of *P2K1* did not show any significant difference 1 or 5 h of treatment with 500 μM of ATP, in comparison to its pattern under mock treatment, i.e., treatment with liquid media (**Figure 1B**). This is a novel result that has not been previously reported. This result suggested that the eATP receptor P2K1 is expressed in the whole seedling, whereas the expression level was stable even after ATP treatment. Based on our data about the stable expression of P2K1 throughout all tissues, we expected that spatial analysis of the expression of eATP-responsive genes would provide further insight into eATP signaling in plants, including the yet- described tissue-specific responses (**Figure 1C**).

### Selection of eATP-responsive marker genes

**Figure 2:**
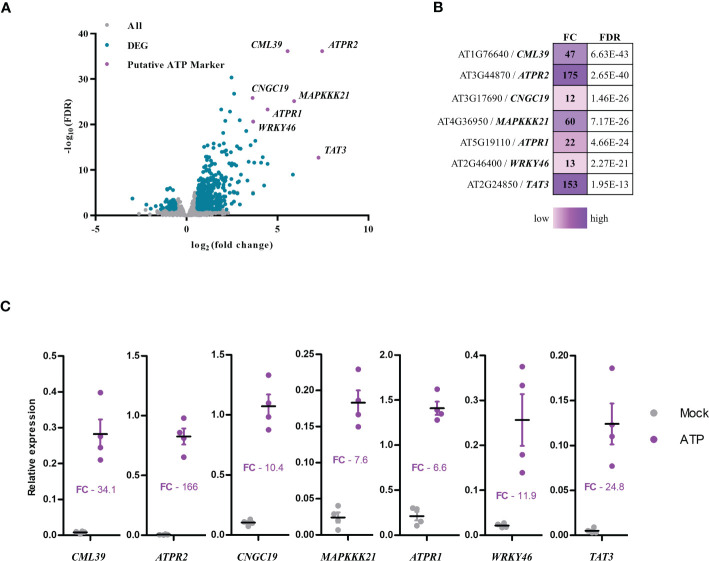
Selection of extracellular ATP (eATP)-sensitive markers. **(A)** Volcano plot of differentially expressed genes in the eATP-induced transcriptome in BioProject accession no. PRJNA494862 (Jewell et al., 2019) plotted as false discovery rate (−log_10_FDR) over fold change (log_2_). **(B)** Table of putative eATP-sensitive markers was shown with fold change (FC) and false discovery rate (FDR) following 500 μM of ATP treatment for 30 min in the transcriptome data. **(C)** Measurement of changes in gene expression of the putative eATP-responsive markers in 7-day-old Col-0 seedlings treated with or without 500 μM of ATP for 30 min. Gene expression was analyzed by RT-qPCR and normalized to PP2A (and SAND with similar results). Individual replicates are shown with mean and standard error bars (*n* = 4). Fisher’s LSD (*P* < 0.05) was used as a statistical cutoff for the comparison of ATP- and mock-treated samples. All the markers were expressed significantly higher following ATP treatment (repeated at least five times with similar results).

Recently, we described eATP-induced transcriptional responses in *Arabidopsis* (Jewell et al., 2019). Putative eATP marker genes were first selected based on fold change (FC) and false discovery rate (FDR, with Bonferroni correction) in the transcriptomics data set (**Figures 2A, B**). Fold change and FDR cutoffs for putative marker genes were 2.5 log_2_ FC and 10 −log_10_ FDR (**Figures 2A, B**)—which reduced the number of candidates to 10. Finally, the genes with less than 1 CPM in mock-treated samples and greater than 10 CPM in ATP-treated samples were considered, leaving seven putative genes as the eATP markers: *CML39* (AT1G76640), *ATPR2* (AT3G44870), *CNGC19* (AT3G17690), *MAPKKK21* (AT4G36950), *ATPR1* (ATG5G19110), *WRKY46* (AT2G46400), and *TAT3* (AT2G24850).

Next, we performed RT-qPCR to validate the seven eATP markers and tested the expression of these genes after ATP treatment by RT-qPCR. After 30 min of ATP treatment, transcripts of *CML39*, *ATPR2*, *CNGC19*, *MAPKKK21*, *ATPR1*, and *WRKY46* were more abundant relative to the mock-treated (liquid media without ATP) samples (**Figure 2C**). These data were nearly comparable to those in the transcriptomics data (**Figure 2B**). For example, the ATP-induced fold change of RT-qPCR *vs*. transcriptomics data was as follows: 34.1 *vs*. 47 for *CML39*, 166 *vs*. 175 for *ATPR2*, 10.4 *vs*. 12 for *CNGC19*, 7.6 *vs*. 60 for *MAPKKK21*, 6.6 *vs*. 22 for *ATPR1*, 11.9 *vs*. 13 for *WRKY46*, and 24.8 *vs*. 153 for *TAT3* (**Figures 2B, C**). These results confirmed the validity of these seven genes as the eATP markers that we selected. While distinct RNA processing and normalization techniques in RNA-seq and RT-qPCR experiments can introduce some variation when comparing their data (Everaert et al., 2017), the results generally agree that these seven genes are significantly and reliably upregulated by eATP treatment as previously described (Jewell et al., 2019).

### Basal expression patterns of eATP marker genes

**Figure 3:**
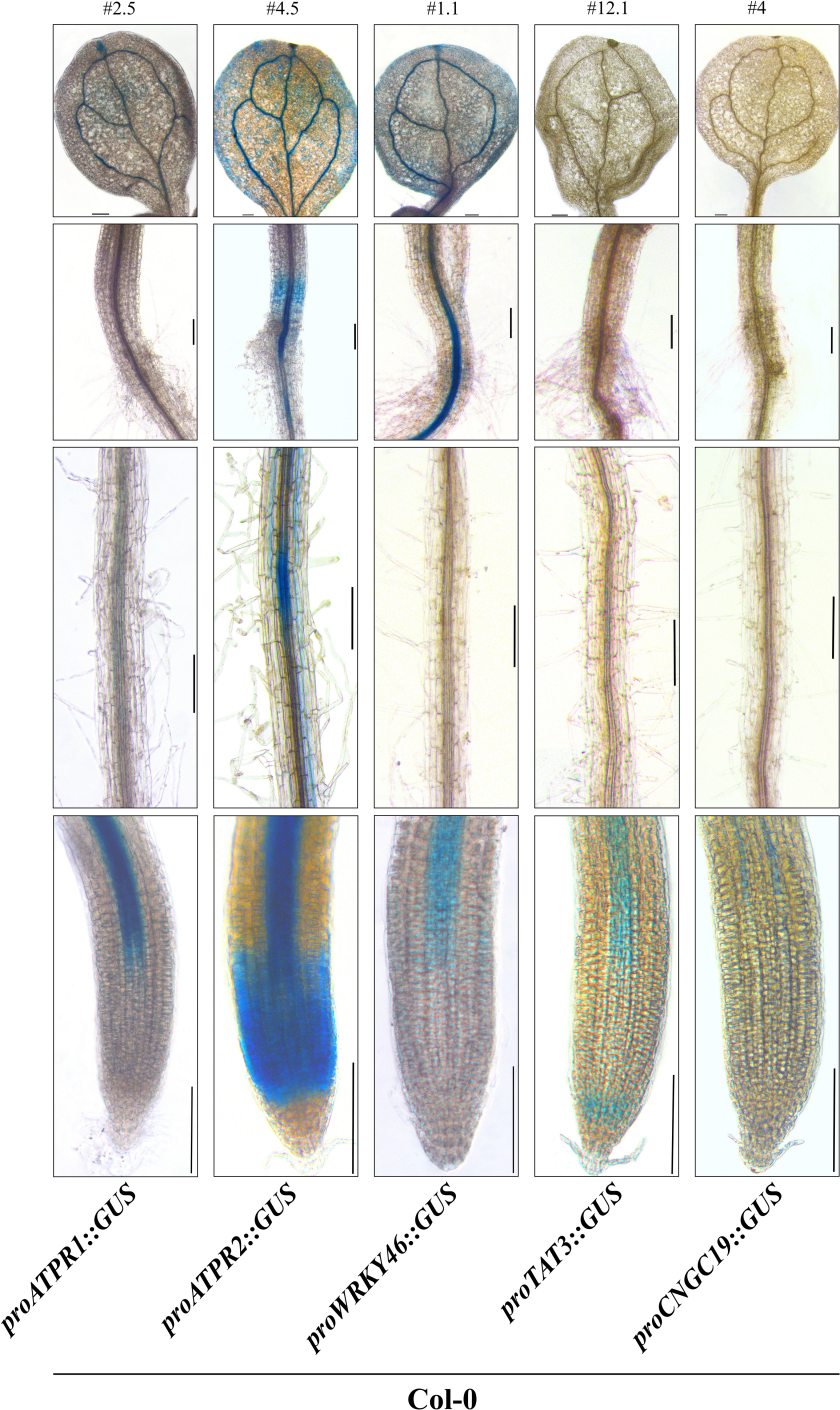
ATP marker genes have a shared pattern of vascular activity. Representative β-glucuronidase (GUS) staining of five eATP marker gene promoter::GUS fusions (*ATPR1*, *ATPR2*, *WRKY46*, *TAT3*, and *CNGC19*) in stably transformed 7-day-old seedlings in the wild-type background. Each line (# above the top panel) is representative of promoter activity observed in at least three independent transformants. Panels from top to bottom: cotyledon, hypocotyl/root junction, distal root, primary root tip. All images were obtained on an inverted phase- contrast microscope (Leica DMI6000). Scale bars = 150 μm.

After generating the eATP marker GUS reporter germplasm, two to five representative lines were selected for each of the promoter/genotype combinations, except for *pCNGC19::GUS* #4, already described (Kugler et al., 2009). We were unsuccessful in cloning the promoter region for *MAPKKK21.* We could create *pCML39::GUS* transgenic plants. However, its GUS staining was highly saturated before ATP treatment. Therefore, these two genes were undesirable for our purpose and excluded from further experiments. First, basal expression (no treatment of any kind) patterns for the reporters in 7-day-old wild-type plants were recorded for a representative line of *pATPR1::GUS* (line #2.5), *pATPR2::GUS* (line #4.5), *pWRKY46::GUS* (line #1.1), and *pTAT3::GUS* (line #12.1) along with *pCNGC19::GUS* #4 (**Figure 3**). Supplemental lines had similar expression patterns and are shown in **Figure S2**. Three of the reporters for *ATPR1*, *ATPR2*, and *WRKY46* had detectable expression in the cotyledon leaf, i.e., the leaf vasculature (**Figure 3**), while two reporters for *ATPR2* and *WRKY46* showed expression in the hypocotyl, near the root junction (**Figure 3**). All five markers were expressed in at least one region of the root tip and the distal primary root vasculature (**Figure 3**). In addition to distal vasculature, *ATPR2* expression was identified throughout root tip tissues approximately corresponding to the primary root meristematic zone, while *TAT3* was also expressed in the meristematic zone that was limited to the most distal portion near or overlapping the stem cell niche (**Figure 3**). No expression was detectable in columella cells.

### eATP-induced reporter expression in a time-dependent manner

To identify an ATP treatment duration maximizing reporter intensity, the reporter line *pCNGC19::GUS* as a representative was treated with 500 μM of ATP for 0–90 h (**Figure S1**). Maximum GUS reporter responses to ATP treatment were identified between 1 and 5 h following treatment (**Figure S1**), and 2 h was selected as the optimal treatment duration for further evaluation. While the average maximal expression among the markers was 2 h post- eATP treatment, minor deviations included *TAT3*, which was highly induced as early as 1 h after treatment, and *ATPR1*, which peaked between 2 and 5 h (**Figure S1**). Next, to provide a comparison of ATP-sensitive promoter activities, the duration of GUS staining was performed identically for each promoter–reporter line. Staining for 1 h at 37°C was selected as the ideal incubation period since it allowed low promoter activity to be detected (e.g., *proCNGC19*), without allowing highly active reporters to become oversaturated (data not shown). It is worth noting that prolonged incubations in GUS staining buffer (e.g., overnight at room temperature) revealed additional expression patterns in some cases. For example, expression of *CNGC19* was detected in the root cap of ATP-treated seedlings but only after prolonged GUS staining (**Figure S1**).

### eATP-induced reporter expression in the root tip

**Figure 4:**
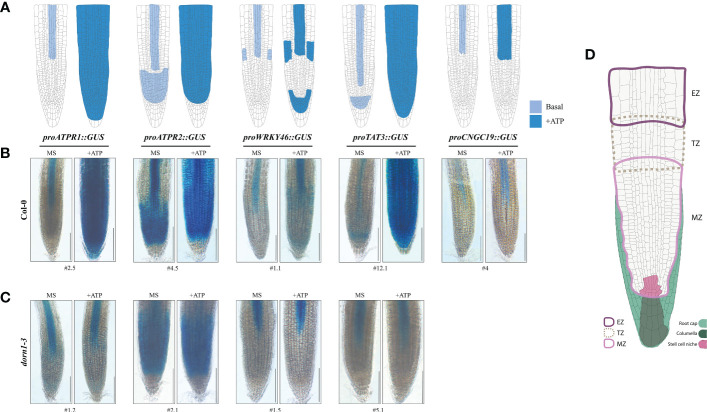
The eATP reporters are activated in the primary root tip and require P2K1. **(A)** Graphical summary of ATP-responsive eATP marker expression patterns in the primary root tip of the promoter::*GUS* reporter lines. **(B, C)** GUS staining in the 7-day-old wild-type or *dorn1-3* mutant plants stably transformed with ATP-sensitive promoter::*GUS* reporters treated with MS (mock) or MS plus 500 μM of ATP (+ATP) for 2 h. *pCNGC19::GUS* was not generated in the *dorn1-3* background. Scale bars = 150 μm. **(D)** The graphic guide for primary root morphology shows the meristematic zone (MZ; solid border in light pink), transition zone (TZ; dotted border in light brown), elongation zone (EZ; solid border in purple), root cap (filled in light green) including the columella (dark green), and the stem cell niche (filled in pink) for reference.

Interestingly, for each of the GUS reporters, ATP-dependent expression was confined to the distal portion of the root vasculature, root tip, or both 2 h after ATP treatment (**Figures 4A, B**). *ATPR1*, *ATPR2*, and *TAT3* shared a similarly remarkable ATP response pattern throughout the root tip, with some notable differences, while the responses for *WRKY46* and *CNGC19* were more subtle. ATP-induced *ATPR1* expression was observed throughout the root meristem zone tissues, including the stem cell niche (SCN) and columella cells, after 60 min (**Figure S2**) and extended to the transition and elongation zones after 2 h (**Figure 4B**; **S2**). Similarly, the induction of *TAT3* expression by ATP occurred throughout the root tip meristem after 60 min and extended into the transition and elongation after 2 h (**Figure 4B**; **S2**). Meanwhile, *ATPR2* expression was already detectable throughout the meristem before ATP treatment and was noticeably induced throughout the primary root transition and elongation zones after ATP treatment, but not in the most distal root tip cell files, in contrast to *ATPR1* and *TAT3* (**Figure 4**). After ATP treatment, *WRKY46* expression increased throughout the distal third of the root vasculature and in the root meristem, where it had been undetectable before ATP treatment (**Figure 4B**). Similarly, the *CNGC19* expression was slightly upregulated in the distal root vasculature (**Figure 4B**) and root tip, with the latter only detectable after prolonged GUS staining (**Figures S1**, **S2**). Overall, all five reporters were responsive to ATP treatment in the root tip of the seedlings.

To determine whether the eATP receptor P2K1 is implicated in the spatial expression patterns of eATP marker genes, we examined the individual GUS reporters in a P2K1 null mutant, *dorn1-3*. Basal expression patterns of ATP-responsive GUS reporters were similar in the *dorn1-3* plants compared with the wild-type plants (**Figures 4B, C**; **S3**). However, some subtle exceptions were observed. For example, basal expression of *ATPR1* in *dorn1-3* induced low levels of expression in approximate primary root SCN and transition zone cell files which were not observed in the wild-type background (**Figure 4**). Meanwhile, *TAT3* was not expressed in the meristem region in *dorn1-3*, which was also contrary to that in the wild-type background (**Figure 4**). Notably, the expression patterns of all GUS reporters in *dorn1-3* remained unaltered upon ATP addition. In conclusion, our results indicate that P2K1 is essential for normal basal and eATP-responsive expressions.

## Discussion

In the present study, we selected the five genes *ATPR1*, *ATPR2*, *WRKY46*, *TAT3*, and *CNGC19* from the transcriptomics data previously reported (Jewell et al., 2019) as the eATP-responsive markers that were highly upregulated upon ATP treatment. Through the use of the GUS reporter system, we discovered that the five eATP marker genes responded significantly to ATP treatment, particularly in the root tip tissue, where the functional P2K1 receptor was essential for their complete response to eATP (**Figure 4**).

*ATPR1* is an uncharacterized gene (annotated as putative dermal glycoprotein-like or aspartyl protease family protein). In an *Arabidopsis* N-myristoylome, *ATPR1* was identified with the myristoylation motif, MGSSLTRLLV (Boisson et al., 2003). In another report, *ATPR1* was identified in cell wall protein isolates (Irshad et al., 2008). To our knowledge, *ATPR1* has not been described in detail, though the *Arabidopsis* eFP browser (https://bar.utoronto.ca/efp_arabidopsis/cgi-bin/efpWeb.cgi) indicates that it is transcriptionally activated by *Pseudomonas syringae* inoculation, salt stress, and methyl jasmonate treatment (Kilian et al., 2007; Winter et al., 2007). Interestingly, our data showed that *ATPR1* is induced by eATP without full dependence on the defense signaling hormones jasmonic acid (JA), salicylic acid (SA), or ethylene (ET) since it still responds to ATP in the signaling mutants of those hormones (**Figure S4**; **Table S3**). Thus, the *pATPR1::GUS* reporter has the potential to be a unique tool for the study of eATP signaling without the full requirement of inputs from plant stress hormones (i.e., JA, SA, and ET).

Prolonged GUS staining demonstrated *WRKY46* promoter activity throughout the root vasculature (**Figure S4**), in agreement with past reports (Ding et al., 2014; Ding et al., 2015; Di et al., 2021). The promoter activity was further upregulated upon ATP treatment (**Figure 4**; **S1**). Previous studies have shown brassinosteroids, drought, and salt stress-induced *WRKY46* expression (Ding et al., 2014; Sheikh et al., 2016; Chen J. et al., 2017), while abscisic acid repressed *WRKY46* expression (Ding et al., 2015). Furthermore, suppression of two apyrase genes, *APY1* and *APY2*, leads to the upregulation of *WRKY46* (Lim et al., 2014)—presumably the result of eATP accumulation in the absence of apyrase-mediated hydrolysis. This report corroborates *WRKY46* as an eATP-responsive marker. Like *ATPR1*, *WRKY46* was induced by eATP independently of signaling pathways associated with JA, SA, and ET (**Figure S4**; **Table S3**), indicating that it can be used as a useful tool for the study of eATP signaling with any confounding influence from these hormones. While it is known that the induction of *WRKY46* expression by SA requires the SA receptor NPR1 (Zhang et al., 2021), eATP was still able to induce *WRKY46* in the *npr1-3* null mutant (**Figure S4**; **Table S3**), suggesting that *WRKY46* activation by eATP is independent of SA. It will be prudent to evaluate the downstream impact of *WRKY46* activation on eATP signaling in the near term.

*ATPR2* is a predicted Farnesoic Acid Methyl Transferase-Like (FAMT-L) gene that has not been the subject of dedicated research. One report found that *ATPR2* expression was induced 24 h following treatment with alamethicin, a membrane-disrupting agent, and in response to herbivory by *Plutella xylostella* (Chen et al., 2003). Although disparate stress signaling pathways may lead to upregulation of *ATPR2*, the requirement of P2K1 for eATP-induced *ATPR2* upregulation (**Figure 4**) could suggest that eATP accumulation after wounding (i.e., 24 h after alamethicin treatment or herbivory) accounts for previous observations. The transcriptional activation of *ATPR2* was found to be dependent on JA signaling (**Figure S4**; **Table S3**), suggesting a possible convergence of ATP- and JA-dependent wound signals in the regulation of *ATPR2*. The *ATPR2* promoter spans 1,180 base pairs upstream of the gene. It is noteworthy that three gene loci, namely, ATG3G44870 (*ATPR2*), ATG3G44880 (*ACCELERATED CELL DEATH1*), and ATG3G44890 (*RIBOSOMAL PROTEIN BL9C*), are arranged in tandem on the third chromosome of *Arabidopsis*. It cannot be ruled out that the intergenic sequence upstream of *ATPR2* may regulate one or more of these genes.

*TAT3* is a putative tyrosine aminotransferase (TAT) and is transcriptionally activated in response to JA and wounding, which depends on the intact JA signaling pathway (Yan et al., 2007; Riewe et al., 2012). Similarly, our data indicate that *TAT3* expression in response to eATP treatment was dependent on JA perception (**Figure S4**; **Table S3**). Interestingly, it has been reported that the expression of *TAT3* in response to JA treatment was dependent on the SA receptor NPR1, based on a reduced fold induction of *TAT3* in the *npr1-1* mutant after JA treatment (Brosché and Kangasjärvi, 2012). In partial agreement, our transcriptomics data (Jewell et al., 2019) showed that differential expression of *TAT3* was reduced in the *npr1-3* null mutant (FC = 46) compared with Col-0 (FC = 153) but remained highly upregulated (**Figure S4**; **Table S3**). Considering the antagonistic relationship between SA and JA, it could be that NPR1 has a greater contribution toward regulating *TAT3* in response to JA, compared with eATP. Notably, eATP-induced *TAT3* response was completely dependent on the JA signaling pathway since the response disappeared in the *coi1* mutant (**Figure S4**; **Table S3**). The reciprocal modulation between signaling pathways linked to other hormones is still not well understood. The *pTAT3::GUS* reporter has the potential to be a valuable tool for exploring the intricate connections between eATP and other plant hormones.

The *pCNGC19*::*GUS* reporter was originally used to demonstrate transcriptional regulation of *CNGC19* by NaCl (Kugler et al., 2009). Interestingly, *CNGC19* was transiently upregulated by salt treatment in the roots at early time points (~30 min) but was only induced in the shoots at later time points, from 6 to 72 hpt (Kugler et al., 2009). In contrast, our data showed that eATP induced the expression of *CNGC19* in the root tip after 1 h of treatment which continued for at least 90 h after treatment (**Figure S1**). In addition, eATP-induced *CNGC19* expression does not require the hormonal signaling pathways JA, SA, or ET (**Figure S4**; **Table S3**). Meanwhile, there was no difference in *CNGC19* expression in the shoots after eATP treatment (**Figure S2**). Similar to eATP treatment, boron starvation was reported to induce *CNGC19* expression in the root tip (Quiles-Pando et al., 2013), along with *CPK28*, another ATP-responsive gene (Choi et al., 2014). In the leaves and roots, *CNGC19* is a permeable channel for Ca^2+^ mobilization in response to herbivory or colonizing fungi (Meena et al., 2019; Jogawat et al., 2020). One of the initial signaling responses to eATP perception is cytosolic Ca^2+^ influx mediated by P2K1 (Choi et al., 2014). Thus, it is possible that the expression of *CNGC19* primes local tissues by increasing sensitivity to Ca^2+^ flux, amplifying any response to a second wave of stimuli (e.g., more damaged cells releasing eATP). Future studies on the spatiotemporal correlation between Ca^2+^ levels and gene expression using *pCNGC19::GUS* would enhance our understanding of the role of *CNGC19*-dependent priming in response to wounding and eATP treatment.

In conclusion, the five eATP-responsive markers we selected—*ATPR1*, *ATPR2*, *WRKY46*, *TAT3*, and *CNGC19*—are highly responsive to eATP in the root tip. These results suggest that the primary root tip is a critically important hub for eATP signaling. The physiological role of P2K1 in the primary root tip has not been extensively explored and requires further investigation. Transcriptional reporters have been a powerful tool for characterizing signaling pathways in plants; auxin is a prime example (Jedličková et al., 2022). Thus, the multigene eATP reporter system we developed can serve as a valuable tool to explore and assess new aspects of eATP signaling in plants.

## Data availability statement

The original contributions presented in the study are included in the article/**Supplementary Material**. Further inquiries can be directed to the corresponding author.

## Author contributions

KT conceptualized the project. KT and JS designed the experiments. JS performed the experiments and prepared the manuscript. KT and JS edited and revised the manuscript. All authors contributed to the article and approved the submitted version.
