# Supplementary material for: A histochemical reporter system to study extracellular ATP response in plants
Source: Front Plant Sci. 2023 Jun 2;14:1183335. doi: 10.3389/fpls.2023.1183335 (PMC10272726; doi:10.3389/fpls.2023.1183335)
Supplement: Supplementary file 1 [file DataSheet_1.pdf]

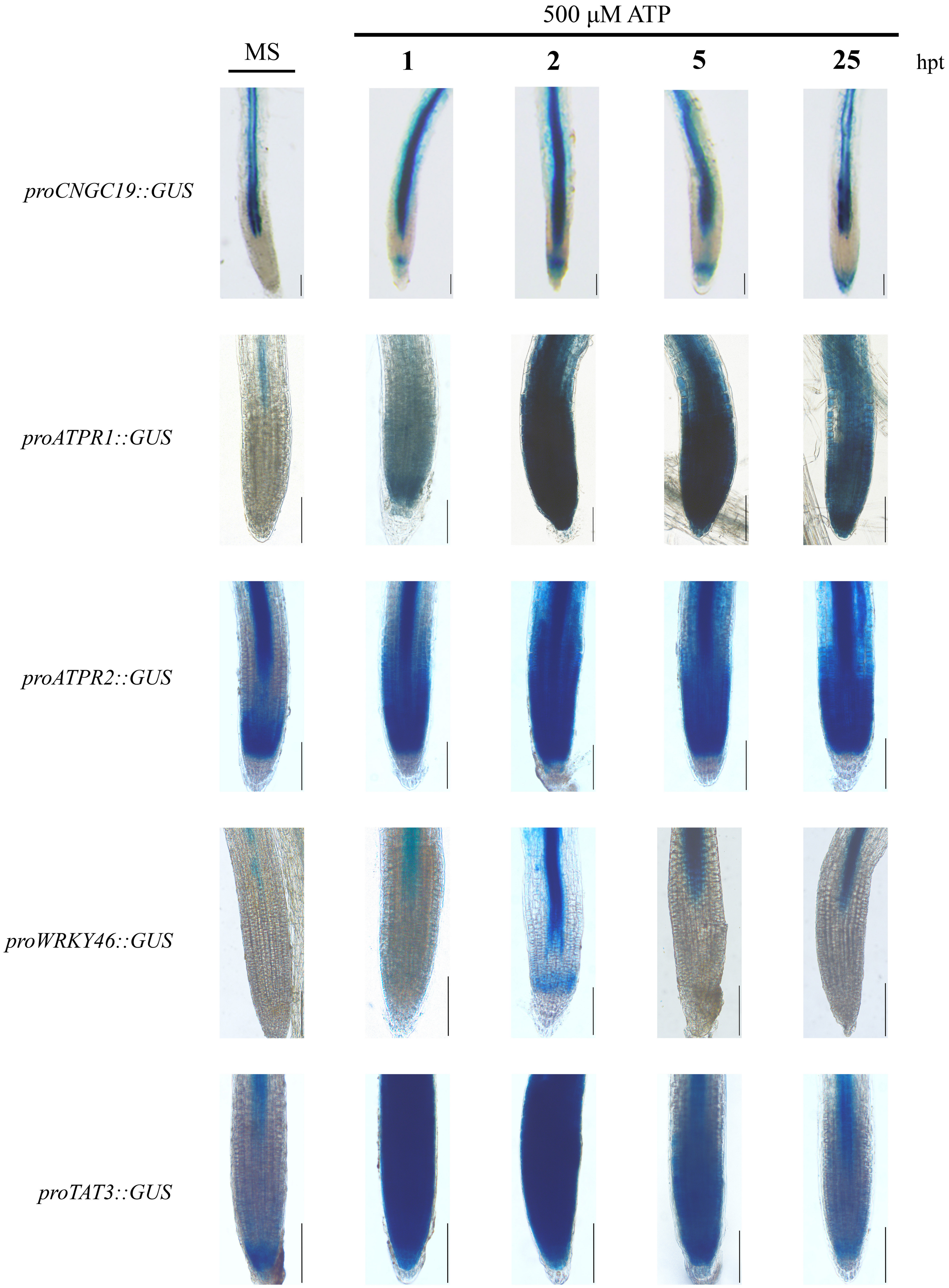

Figure S1. Time-dependent response of GUS reporters after ATP treatment
Time course expression of the eATP reporters at various hours post-treatment (hpt) with 500 μM ATP. After harvesting, the seedlings were incubated in 2 mM X-Gluc at room temperature overnight. In later experiments, the staining concentration and duration were reduced to increase the resolution of eATPsensitive promoter activity across all promoters compared to the baseline, which consequently prevented the detection of root meristem and root cap ATP responses in pCNGC19::GUS (as in Figure 4). Scale bar = 150 μm.

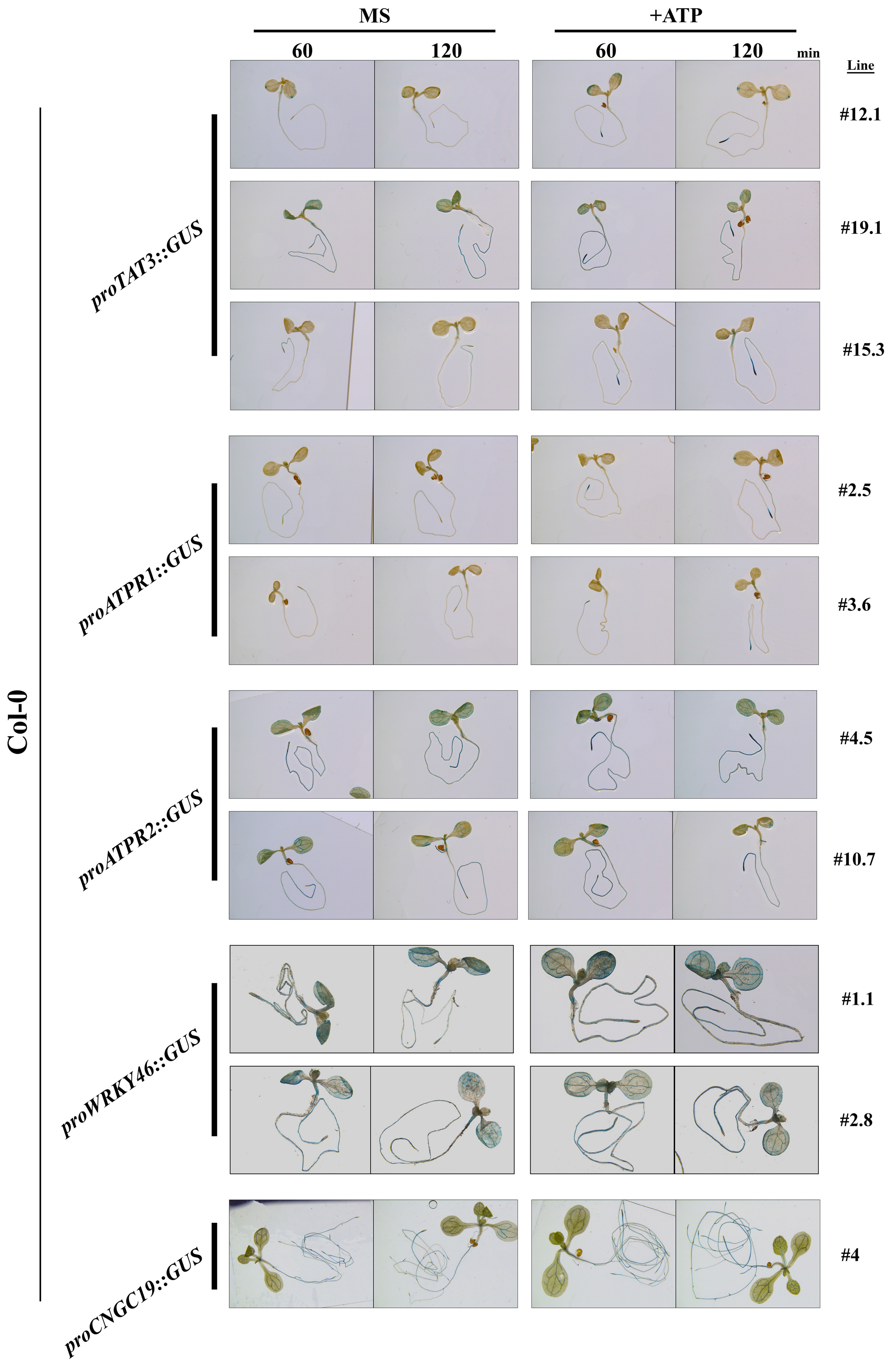

Figure S2. All eATP marker promoter::GUS germplasm in Col-0
The seedlings (7- or 10-d-old) of the eATP-responsive promoter::GUS reporter lines in the wildtype background were treated with MS or MS plus 500 µM ATP for 60 or 120 min. Histochemical GUS staining was done overnight (~10 h) at room temperature.

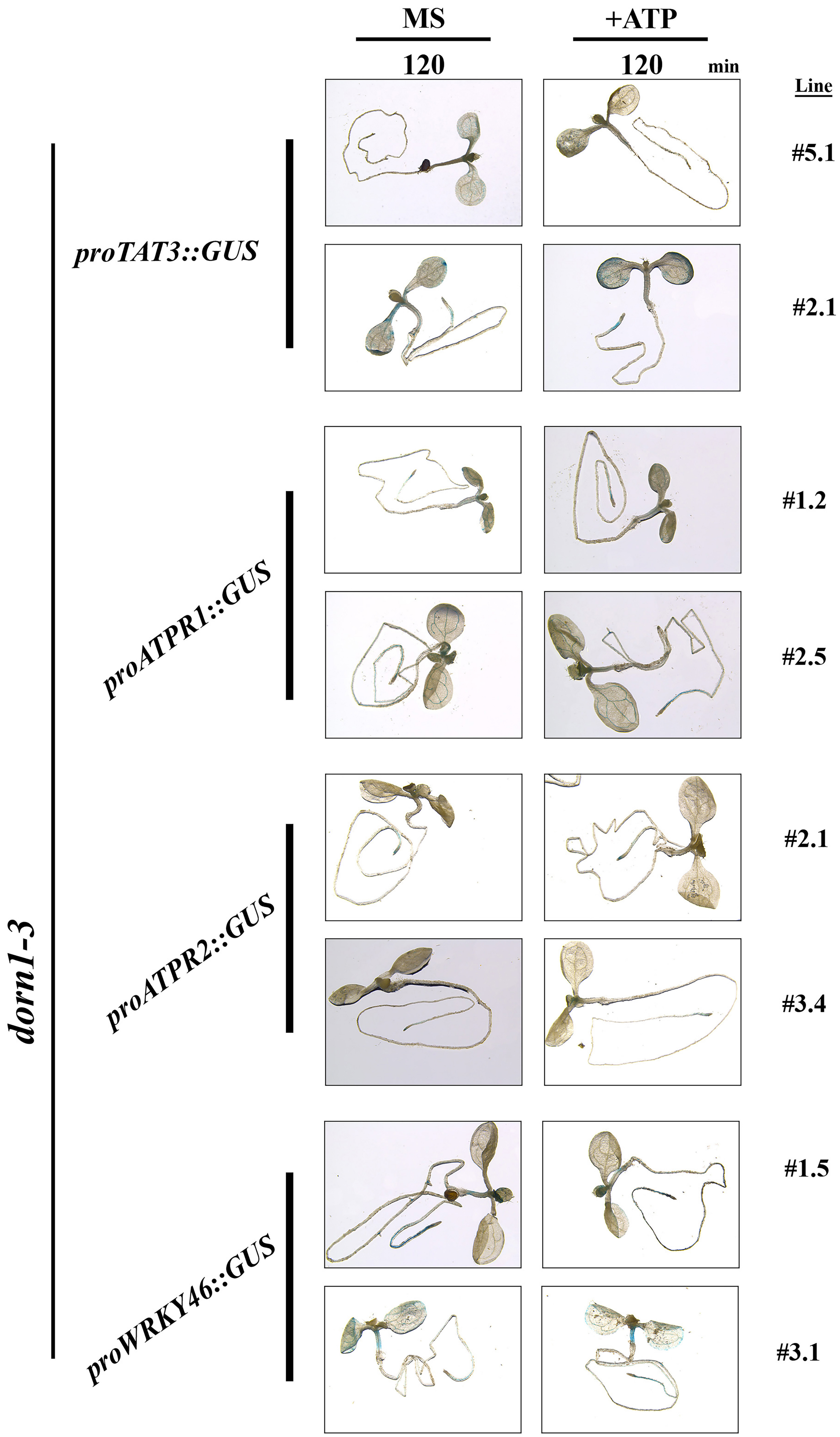

Figure S3. All eATP marker promoter::GUS germplasm in dorn1-3

The seedlings (7-d-old) of the eATP-responsive promoter::GUS reporter lines generated in the dorn1-3 mutant background were treated with MS or MS plus 500 μM ATP for 120 min. Histochemical GUS staining was done overnight (~10 h) at room temperature.

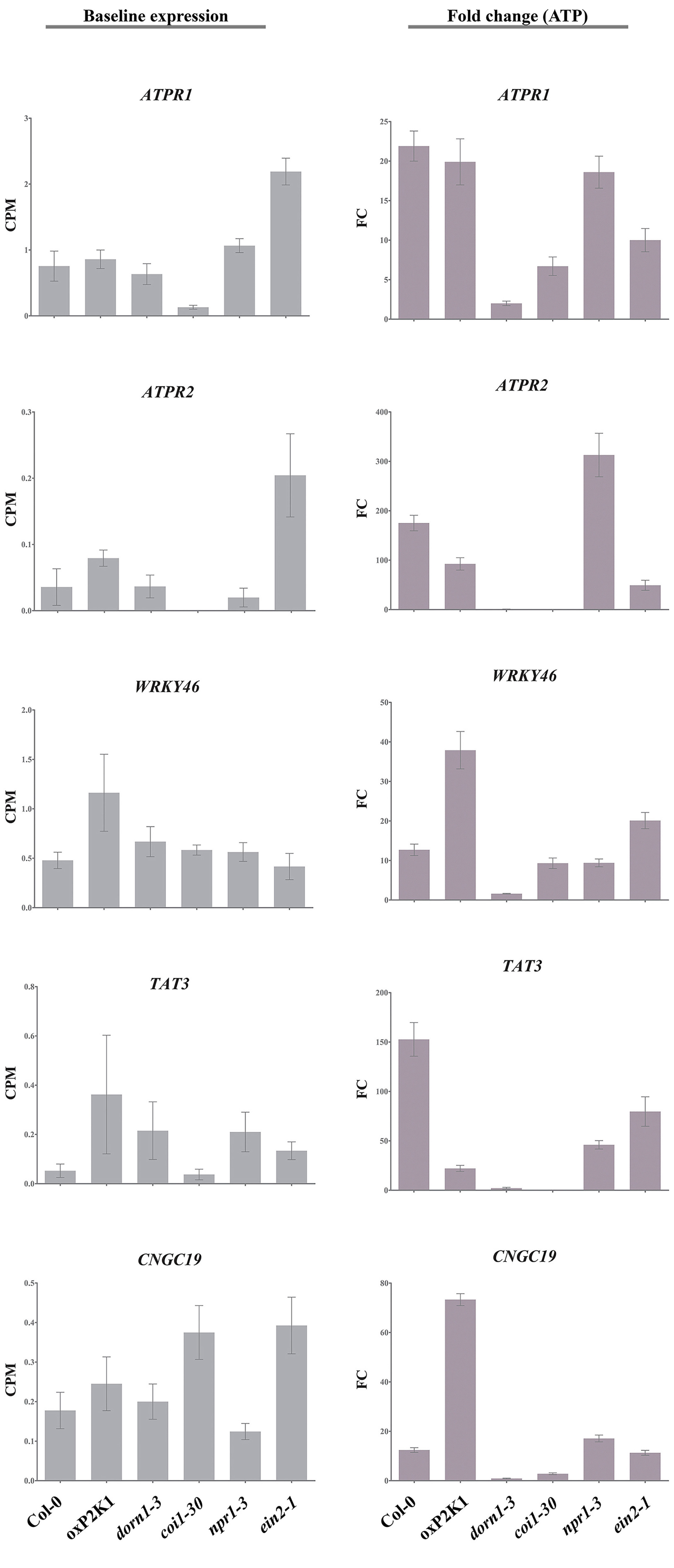

Figure S4. ATP Marker Expression by Genotype

Baseline expression (untreated) of each gene is shown (left, gray) for the respective genotypes in transcript counter per million read (CPM). Fold change (FC, right, purple) in marker gene expression after ATP treatment in different genotypes and knockout mutants of stress-related plant hormones. The data were reproduced from the transcriptomic dataset by Jewell et al. (2019). Numerical data were shown in Table S3.

Primer name	Sequence (5'---3')	Note	Ref	Use
**Construction**				
proTAT3 Forward	TTTAAGCTTCTGGATATGGCGTTTCTCTA	***Hind*III**	**This study**	**pCAMBIA1305.2**
proTAT3 Reverse	TTTCCATGGTGATCTTTCTGTCTTGCGTTTG	***Nco*I**		
proWRKY46 Forward	TTTAAGCTTCTGGTAACGGTGGACTAATG	***Hind*III**		
proWRKY46 Reverse	TTTCCATGGCTTTGCATTCACTTCAGAAAATT	***Nco*I**		
proACD1 Forward	TTTAAGCTTGAGAGATAATGTTTCCCTTATCC	***Hind*III**		
proACD1 Reverse	TTTCCATGGGTCTCTCTTTTAACTCCTGTCTT	***Nco*I**		
proATPR1 Forward	TTTAAGCTTAGTGCTGAGTAGACTT	***Hind*III**		
proATPR1 Reverse	TTTCCATGGCTCTATTATATTCAGGTTATAA	***Nco*I**		
**RT-qPCR**				
qPP2A Forward	TAACGTGGCCAAAATGATGC		**(Czechowski et al., 2005)**	**RT-qPCR**
qPP2A Reverse	GTTCTCCACAACCGCTTGGT			
qCML39 Forward	TGCAGAGGAGCTTAAGAAGAGTTTTA		**(Jewell et al., 2019)**	
qCML39 Reverse	CCTCGGCTTCTTCATCAGACA			
qCNGC19 Forward	CATCGAGTGCAAGGAGCTATAA		**(Jewell et al., 2019)**	
qCNGC19 Reverse	GGAGTTGTCTCTTTCGGTATCTC			
qMPKKK21 Forward	TTCCCGGTTGGAATTCGGTT		**(Jewell et al., 2019)**	
qMPKKK21 Reverse	AATCACACGACACCGACCAA			
qTAT3 Forward	GCACGAAGCTAGTTAGTGAGGA		**(Dombrecht et al., 2007)**	
qTAT3 Reverse	CACTGATTCGTCGGTTCCTA			
qWRKY46 Forward	TGAGATTGAGAACGGTGTGTG		**(Jewell et al., 2019)**	
qWRKY46 Reverse	CCCTGATGTTGCAGGAGAAA			
qACD1 Forward	TGCTCCACAAGCGGATGAAT		**(Jewell et al., 2019)**	
qACD1 Reverse	TCCAACCGTGACCTTAAAGGAG			
qATPR1 Forward	GGGTTCTACGGTGCGAATAC		**(Jewell et al., 2019)**	
qATPR1 Reverse	GTCGATAAACGCCAAACACATC			

Table S1. Primers used in this study

Arabidopsis germplasm/background	Vector	Representative lines	Supplemental lines	Source
proP2K1::*GUS*/Col-0	pMDC	#1	N/A	(Cho et al., 2017)
proCNGC19::*GUS*/Col-0	pGPTV-HPT	#4	N/A	(Kugler et al., 2009)
proATPR2::*GUSPlus*/Col-0	pCAMBIA1305.2	#4.5	#10.7	This paper
proATPR1::*GUSPlus*/Col-0	pCAMBIA1305.2	#2.5	#3.6	This paper
proTAT3::*GUSPlus*/Col-0	pCAMBIA1305.2	#12.1	#15.3, #19.1	This paper
proWRKY46::*GUSPlus*/Col-0	pCAMBIA1305.2	#1.5	#2.8	This paper
proATPR2::*GUSPlus*/*dorn1-3*	pCAMBIA1305.2	#2.1	#3.4	This paper
proATPR1::*GUSPlus*/*dorn1-3*	pCAMBIA1305.2	#1.2	#2.5	This paper
proTAT3::*GUSPlus*/*dorn1-3*	pCAMBIA1305.2	#5.1	#2.1	This paper
proWRKY46::*GUSPlus*/*dorn1-3*	pCAMBIA1305.2	#1.5	#3.1	This paper

Table S2. Germplasm used in this study

Name	Col-0			oxP2K1			*dorn1-3*			*coi1-30*			*npr1-3*			*ein2-1*		
	CPM	FC	FDR	CPM	FC	FDR	CPM	FC	FDR	CPM	FC	FDR	CPM	FC	FDR	CPM	FC	FDR
ATPR1	0.755	21.9	4.66E-24	0.634	19.9	3.01E-25	0.859	2	1.00E+00	0.131	6.7	3.82E-06	1.066	18.6	1.78E-22	2.19	10	1.71E-13
CNGC19	0.178	12.4	1.46E-26	0.2	73.3	2.80E-59	0.245	0.9	1.00E+00	0.375	2.8	9.84E-05	0.124	17.1	3.62E-30	0.393	11.3	1.28E-29
WRKY46	0.478	12.7	2.27E-21	0.668	37.9	1.04E-74	1.162	1.6	1.00E+00	0.583	9.3	5.32E-16	0.563	9.4	3.90E-17	0.416	20.1	1.82E-24
ATPR2	0.036	175.2	2.65E-40	0.037	92.5	7.97E-40	0.079	0.9	1.00E+00	0.007	0	1.00E+00	0.02	312.9	1.08E-44	0.204	49.2	1.08E-28
TAT3	0.052	152.7	1.95E-13	0.215	22.1	2.53E-08	0.362	2.1	1.00E+00	0.037	0.1	3.79E-01	0.21	46	1.11E-09	0.134	79.7	4.78E-10

Table S3. ATP Marker Expression by Genotype
Baseline (untreated plants) expression in counts per million (CPM), Fold change (FC), and false discovery rate (FDR) for ATP-responsive gene expression in different genotypes as reported in the transcriptome published by Jewell et al. (2019). Each marker gene's fold change among genotypes is arranged in a sliding color scale (green = highest, white = lowest).
